# Effects of physical activity interventions using wearables to improve objectively-measured and patient-reported outcomes in adults following orthopaedic surgical procedures: A systematic review

**DOI:** 10.1371/journal.pone.0263562

**Published:** 2022-02-15

**Authors:** Hiral Master, Jordan A. Bley, Rogelio A. Coronado, Payton E. Robinette, Daniel K. White, Jacquelyn S. Pennings, Kristin R. Archer

**Affiliations:** 1 Department of Orthopaedic Surgery, Center for Musculoskeletal Research, Vanderbilt University Medical Center, Nashville, Tennessee, United States of America; 2 Vanderbilt Institute of Clinical and Translational Research, Vanderbilt University Medical Center, Nashville, Tennessee, United States of America; 3 Department of Physical Medicine and Rehabilitation, Osher Center for Integrative Health, Vanderbilt University Medical Center, Nashville, Tennessee, United States of America; 4 Department of Physical Therapy, University of Delaware, Newark, Delaware, United States of America; Linneaus University, SWEDEN

## Abstract

**Objective:**

To synthesize evidence on physical activity interventions that used wearables, either alone or in combination with education or rehabilitation, in adults following orthopaedic surgical procedures.

**Methods:**

PubMed, CINAHL, PsycINFO and EMBASE were searched for randomized controlled trials of wearable-based interventions from each database’s inception to August 2021 in patients undergoing orthopaedic surgery. Relevant outcomes included physical activity, physical function, pain, psychological distress, or general health. PEDro scale scoring ranges from 0 to 10 and was used to appraise studies as high (≥7), moderate (5–6), or poor (<5) quality.

**Results:**

Of 335 articles identified, 6 articles met eligibility criteria. PEDro scores ranged from 2 to 6, with 3 studies of moderate quality and 3 of poor quality. Studies included patients undergoing total knee (number; *n* = 4) or total knee or hip (*n* = 1) arthroplasty and lumbar disc herniation surgery (*n* = 1). In addition to wearables, intervention components included step diary (n = 2), motivational interviewing (*n* = 1), goal setting (*n* = 2), tailored exercise program (*n* = 2), or financial incentives (*n* = 1). Interventions were delivered in-person (*n* = 2), remotely (*n* = 3) or in a hybrid format (*n* = 1). Intervention duration ranged from 6 weeks to 6 months. Compared to controls, 3 moderate quality studies reported greater improvement in steps/day; however, 1 moderate and 2 poor quality studies showed no between-group difference in physical function, pain, or quality of life. No serious adverse events related to the use of wearable were reported.

**Conclusions:**

The effects of physical activity interventions using wearables, either delivered in-person or remotely, appear promising for increasing steps per day after joint arthroplasty; however, this finding should be viewed with caution since it is based on 3 moderate quality studies. Further research is needed to determine the therapeutic effects of using wearables as an intervention component in patients undergoing other orthopaedic surgical procedures.

**Trial registration:**

PROSPERO Registration Number: CRD42020186103

## Introduction

Orthopaedic surgeries, such as joint arthroplasty and spine arthrodesis, are commonly performed in the United States and associated with high costs for managing musculoskeletal disorders [1–3]. Despite improvements in patient-reported pain and function following orthopaedic surgery, physical activity often remains unchanged [4–8]. Physical activity is defined as any energy expenditure above the resting level and includes a range of activities that patients perform at home or in the community [9]. Low levels of physical activity are associated with adverse health outcomes, including increased risk of functional limitation, disability, cardiovascular disease, diabetes, and mortality [10–15]. Therefore, promoting physical activity is critical for patients after orthopaedic surgery to optimize their recovery trajectory and overall post-surgical health.

Newer technologies, such as commercially available wearables (e.g., pedometers), can be employed to promote physical activity and combined with self-monitoring and patient-centered goal-setting strategies [16]. Several systematic reviews on wearable-based physical activity interventions suggest beneficial effects in healthy adult and in patients with chronic conditions, including chronic obstructive pulmonary disease, cancer, arthritis, stroke and obesity [17–22]. For community dwelling adults with or without chronic disease, physical activity interventions that leverage wearables have resulted in increased steps per day (Standardized Mean Difference (SMD) ranged from 0.24 to 0.51) and time spent in moderate to vigorous intensity physical activity (SMD ranged from 0.27 to 0.43) [17, 19].

The efficacy of physical activity interventions using wearables is well-established in adults with musculoskeletal disorders such as arthritis and low back pain [20, 21]. Mansi et al. found moderate intervention effects on steps per day in adults with musculoskeletal disorders (mean increment of 1950 steps per day relative to baseline) [20]. Most of the studies included in prior systematic reviews focused on adults with non-operatively managed musculoskeletal conditions. The therapeutic effects of these interventions on health outcomes have not been comprehensively summarized in adults with musculoskeletal disorders who are managed surgically with common procedures such as joint arthroplasty or spine arthrodesis. Studying these populations can provide clinical benefit since strategies to address low levels of physical activity can be feasibly integrated into postoperative management with the potential to improve postoperative outcomes [23–26]. Further, it is not known whether the effects of these interventions vary based on delivery procedure, namely in-person versus remote (e.g. telephone or video calls). Investigating the effects of physical activity interventions that use wearables by delivery procedure is essential since remote interventions are increasingly utilized within an evolving healthcare environment [27, 28] and to increase healthcare access [29].

The aim of this systematic review was to synthesize the evidence on physical activity interventions that used wearables, either stand-alone or in combination with education or rehabilitation, in orthopaedic surgical populations. Additionally, this review examined the efficacy/effectiveness of these interventions on outcomes such as physical activity, physical function, pain, psychological distress, and general health. The findings of this review can inform postoperative management strategies to promote recovery following orthopaedic surgery.

## Materials and methods

### Study registration and reporting

This study was prospectively registered in an international database of systematic reviews in health and social care (registration number CRD42020186103; https://www.crd.york.ac.uk/prospero/). Reporting of this systematic review followed Preferred Reporting Items for Systematic Reviews and Meta-analyses (PRISMA) guidelines.

### Eligibility criteria

PICOS (Participants, Interventions, Comparator, Outcomes and Study design) approach was utilized to guide this systematic review.

#### Participants

Studies involving adults who underwent orthopaedic surgical procedures (e.g. arthroplasty or arthrodesis) to manage musculoskeletal disorders were included in the systematic review. Studies involving participants with primary or comorbid conditions that may impede participation in physical activity (e.g., carcinoma or neurological disorders) were excluded.

#### Intervention

Interventions that used wearable technology such as Fitbits or pedometers as a primary component of the intervention and either as a stand-alone or in combination with education or rehabilitation (i.e., physical therapy or cognitive and behavioral) programs were included. No restriction was placed on the healthcare professional delivering the intervention.

#### Comparison

No limit was placed on the type of comparison group as long as the effect of the intervention (described in previous section) could be determined. Comparison groups could include no treatment, placebo or sham groups, wait-and-see approaches, usual/standard care, and other types of intervention that did not involve the direct delivery of a physical activity intervention that used wearables.

#### Outcomes

The primary outcomes of interest in this review were physical activity, physical function, pain, psychological distress, and general health. Physical activity could be assessed using either objective or patient-reported measures. Physical activity was quantified as steps per day and/or time spent in different intensities of physical activity. Physical function could be assessed using either patient-reported measures or performance-based tests, while pain, psychological distress, and general health could be assessed with patient-reported measures.

#### Study design

The beneficial effects of physical activity are well-established in patients with musculoskeletal pain [10, 20]. Thus, this review was limited to published pilot or fully powered, randomized controlled trials as we examined the feasibility or efficacy/effectiveness of physical activity interventions that incorporate wearable devices. Randomized controlled trials could include parallel or cross-over designs. We excluded all non-randomized or quasi-experimental study designs. Information from book chapters, conference abstracts or proceedings, opinions and commentaries, and previous reviews were also excluded.

### Data sources and searches

PubMed, EMBASE, Cumulative Index to Nursing and Allied Health Literature (CINAHL), and PsycINFO were searched electronically from each database’s inception to August 2021. Only articles published in English were included in the search and no limit was placed on publication date. Search terms included a combination of keywords for musculoskeletal conditions managed surgically and for physical activity intervention types (S1–S4 Tables). Where indicated, MeSH terms or major headings were used within each database. Reference lists of relevant articles were reviewed to identify articles not included within the electronic search. Additionally, a content expert (DKW) was consulted to confirm the final list of selected papers.

### Study selection

All study records identified from the electronic and hand search were imported into Endnote X9 for Windows (Clarivate Analytics, Philadelphia, PA). After duplicates were removed, two independent reviewers (JAB and PER) screened the title and abstract of all studies. Articles not considered relevant based on title and abstract review were excluded. In cases where more information was needed, full texts of articles were screened. Relevant studies identified after title, abstract, and full-text review were compared by the two reviewers and disagreements regarding final eligibility were resolved by consensus. If necessary, a third researcher (HM) was consulted.

### Data extraction

One reviewer (HM) extracted data from each article using a standardized extraction form. Extracted data included study details (author, year, sample size, country), participant characteristics (i.e., surgical procedure, age, and gender), description of components given to control or comparator group, outcome measures, main study results at all follow-up time-points, and adverse events. Additionally, characteristics of how the intervention was developed and delivered (i.e., type of wearable used, mode of delivery, behavioral models used to design the intervention, duration of the program) were extracted. Accuracy of data extraction was verified by a second reviewer (JAB).

### Risk of bias (quality) assessment

Risk of bias assessment was performed using the Physiotherapy Evidence Database (PEDro) scale [30, 31]. The PEDro scale is a reliable and valid measure of the quality of intervention trials. The PEDro scale includes 11 questions on eligibility criteria, participant characteristics, randomization, blinding, statistical analysis, and outcome measures. Each question is rated as Yes or No based on whether the information was reported in the manuscript. Ten of the 11 items are summed for a total score, with higher scores indicating lower risk of bias. Scores of 7 or more, 5 or 6, and less than 5 were considered as high, moderate, and poor quality, respectively [32, 33]. Three reviewers (HM, JAB and PER) independently graded the risk of bias of included studies using the PEDro scale. However, if the trials/studies were listed in the PEDro database (https://www.pedro.org.au/), those scores were used in this review.

### Strategy for data synthesis and analysis

The characteristics of an intervention and effects on outcome measures were qualitatively summarized in this review. The data on physical activity was summarized as steps per day or time (minutes per week) spent in moderate-to-vigorous physical activity (MVPA). If the study presented data for time spent in MVPA per day, these data were converted to minutes per week by multiplying by 7. This strategy was employed to facilitate the interpretation in terms of current Physical Activity Guidelines for Americans [10]. Based on the outcomes included in the review, physical function was summarized as time needed to complete the Timed Up and Go test (TUG), 6-minute walk test (6-MWT) and 4-meter walk test. Patient-reported measures of EuroQol-5 (EQ-5D), Knee Injury and Osteoarthritis Outcome Score (KOOS), Western Ontario and McMaster Universities Osteoarthritis Index (WOMAC), and Short Form survey (SF-36) were used to assess physical function, pain, psychological distress, and general health as appropriate. Pain and disability were assessed using McGill Pain Questionnaire and Oswestry Disability Index, respectively, in participants who underwent spine surgery. Given the variability in outcome time-points, meta-analysis was not performed. All outcomes measured in the intervention and control groups were summarized using means, standard deviation or 95% confidence interval (CI). Further, within-group and between-group difference in the outcome measures in each of the studies included in this review were reported using means and 95% CI or p-values or effect sizes such as Cohen’s d.

## Results and discussion

### Search results

**Fig 1:**
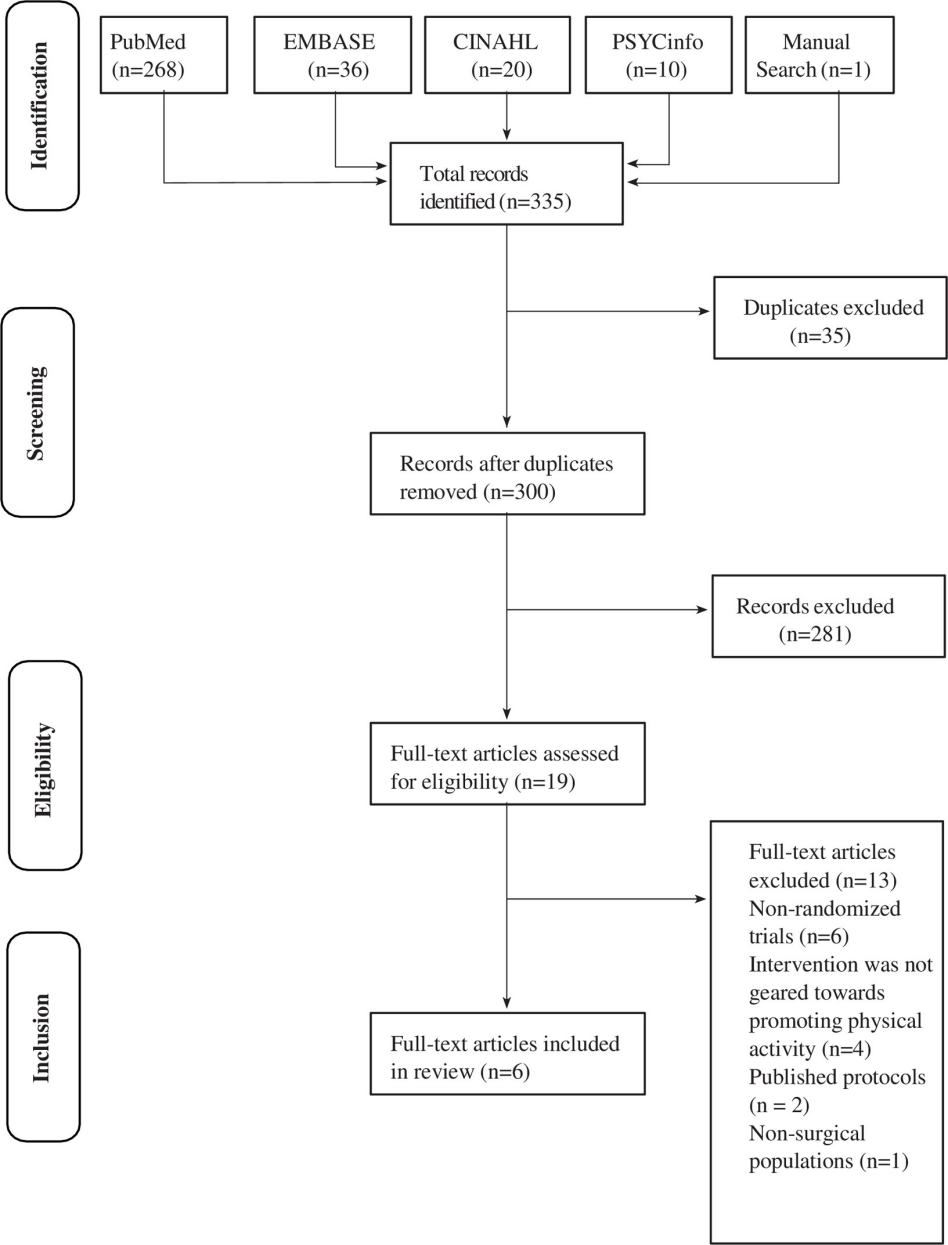
Flow chart of studies included in this review.

A total of 335 articles were identified through search strategy employed for this review (S1–S4 Tables). After removing 35 duplicate articles, 300 unique titles and abstracts were screened. Of these, 281 articles were excluded since they were not considered relevant based on title and abstract, e.g., did not fit condition or intervention criteria (*n* = 235), animal research (*n* = 2), protocol, meeting notes or systematic review articles (*n* = 42) and non-English articles (*n* = 2). Nineteen full-text articles were assessed for eligibility. Of these, 13 articles were excluded; reasons for exclusion included non-randomized trials (*n* = 6), the intervention was not geared towards promoting physical activity using wearable technology (*n* = 4), published protocols (*n* = 2), and non-surgical populations (*n* = 1). Six articles met eligibility criteria (Fig 1).

### Sample characteristics

Five studies included patients undergoing total knee arthroplasty (*n* = 406), one study included patients undergoing total knee or hip arthroplasty (*n* = 95), and one study included patients undergoing surgery for lumbar disc herniation (n = 67). A total of 568 participants were included in this review [34–39] (Table 1). The average age range of the samples across studies reporting age was 64 to 67 years. The samples included a total of 263 men and 305 women. Four (67%) studies were conducted in the United States [34–37].

**Table 1:** Articles included in systematic review.

Study name	Sample size	Surgery and characteristics	inclusion/exclusion criteria	Study length	Treatment for intervention and control groups.	Outcomes and time-points
Aldemir et al, 2021 [39]	Total (N = 67) Intervention (n = 33) Control (n = 34)	Lumbar Microdiscectomy Women: 51%	Inclusion: (i) scheduled for standard lumbar discectomy using a microsurgical technique, (ii) no vision or hearing deficits, (iii) no speech impairment or communication problems, (iv) no mental health problems, (v) literate and owns a cellular phone. Exclusion criteria: (i) 70 years of age and above, (ii) unable to speak, read or write in Turkish, (iii) physical and cognitive problems to the level of not being able to use a cell phone, (iv) multiple Lumbar disc hernia surgeries and (v) presence of joint, ligament or vascular diseases to the point of not being able to walk.	3 Mo	The intervention group received a pedometer along with the user’s manual and, a 12-week record chart prior to surgery. Starting from 3 weeks post-surgery, this group receive a 12 weekly phone calls. During the weekly phone calls, participant’s physical activity for previous week and walking plan for subsequent week would be discussed. Participants in intervention and control groups received 20-to-30-minute face-to-face interview prior to surgery, and at 3-weeks, 1-, 2- and 3-months post-surgery. During the interview, pain, disability and quality of life were assessed. For the intervention group, participant’s pain level during 10-minute walk test would be evaluated during face-to-face interview at 3 weeks post-surgery.	Pain (McGill Pain Questionnaire), Disability (Oswestry Disability Index) and Quality of Life (SF-36) were assessed before, and at 3-weeks, 1-, 2- and 3-months post-surgery. Steps per day, walking duration and distance were assessed using pedometer (brand unknown) at 1-, 2- and 3-months post-surgery.
Christiansen et al, 2020 [34]	Baseline (N = 43) Intervention (n = 20) Control (n = 23)	Total Knee Arthroplasty (TKA) Mean Age: 67±7 years Women: 53%	Inclusion: (i) undergoing unilateral TKA (ii) age >45 years (iii) interested in increasing physical activity Exclusion: (i) scheduled to undergo contralateral TKA or lower extremity surgery within 6 months (ii) presence of co-morbidity that impedes activity participation e.g. unstable angina	12 Mo	The intervention group received a Fitbit Zip, weekly steps/day goal from a physical therapist (in-person), and monthly follow-up phone calls from a research assistant (for 6 months) to promote physical activity. In addition, the intervention group was provided with the same standard outpatient physical therapy as that provided to the control group.	Steps per day and time in MVPA, i.e., minutes per week, both were assessed using triaxial accelerometer (Actigraph GT3X) at baseline (after surgery), Discharge from physical therapy, 6 Mo, and 12 Mo after discharge from physical therapy
Losina et al, 2018 [35]	Total (N = 202) Control (n = 51) Health Coaching (n = 49) Financial Incentives (n = 50) Coaching + Financial Incentives (n = 52)	Total Knee Arthroplasty (TKA) Mean Age: 67±7 years Women: 57%	Inclusion: (i) undergoing unilateral TKA (ii) age ≥40 years (iii) English speaking Exclusion: (i) no internet access on regular basis (ii) scheduled to undergo contralateral TKA or surgery within 6 months (iii) presence of co-morbidity that impedes activity participation e.g. Parkinson’s disease	6 Mo	Participants in intervention group received Fitbit Zip, health coaching and financial incentives received 14 calls (weekly for weeks 2 to 5 and biweekly for weeks 7–24) by research staff trained in motivational interviewing techniques. Open ended questions were used to generate goals with participants. Further, participants were eligible to earn up to $305 over 6 months given if they completed logs or increased their goals. Participants in attention control group also received 14 calls (same schedule) to convey general health information and counsel on general aspects of recovery and rehabilitation.	Steps per day and time in MVPA, i.e., minutes per week, both were assessed using triaxial accelerometer at baseline (after surgery), 3 Mo, and 6 Mo after surgery
Paxton et al, 2018 [36]	Total (N = 45) Intervention (n = 22) Control (n = 23 control)	Total Knee Arthroplasty (TKA) Women: 45%	Inclusion: (i) undergoing unilateral TKA (ii) age 50 to 75 years	3 Mo	Intervention group received real-time activity feedback from Fitbit Zip, weekly action planning phone calls, and monthly in-person group support meetings over 3 Mo. The control group received standard of care post-TKA and weekly phone meetings to monitor participants’ health status, but without physical activity feedback and face-to-face group meetings.	Steps per day was assessed using triaxial accelerometer (Actigraph GT3X) and physical function was assessed using TUG, 6-minute walk and 4 meter walk test at 6–8 weeks, and 4.5 to 5 Mo from surgery
Smith et al, 2019 [37]	Total (N = 60) Intervention (n = 30) Control (n = 30)	Total Knee Arthroplasty (TKA) Mean Age: 64±9years Women: 56%		4 Mo	Intervention group received fitness tracker + 4 Mo tailored home-based exercise program (included tailored resistance and aerobic training) + weekly phone calls from the study exercise physiologist (to monitor compliance, assess the patient’s progress, and modify the exercise prescription as needed). Control group received 16-week tailored home-based exercise program for exercise prescription from the American College of Sports Medicine	6-minute walk test, WOMAC, SF-36, at 2 Mo & 4 Mo from surgery
Van der Walt et al, 2018 [38]	Total (N = 163) Intervention (n = 81) Control (n = 82)	Total Knee Arthroplasty (TKA) and Total Hip Arthroplasty (THA) Mean Age: 64±9years Women: 50%	Inclusion: (i) undergoing primary TKA or THA Exclusion: (i) presence of rheumatoid arthritis or other inflammatory diseases, (ii) undergoing THA after an acute femoral fracture and (iii) unable to contact within 2 weeks of surgery	6 Mo	Participant in the intervention group were allow to see the steps on Garmin Vivofit 2 and received daily step goals until 6 weeks post surgery. However, those in the control group were not allowed to see the steps on Garmin Vivofit 2 and received no steps goals.	Steps per day was assessed using Garmin Vivofit 2 at preoperative, 6 weeks and 6 Mo after surgery. Patient-reported measures such as EQ-5D and KOOS were used to assessed pain, physical function and quality of life at preoperative and 6 Mo after surgery

Mo = months; MVPA = moderate-to-vigorous activity; ROM = range of motion; TUG = Time UP and Go Test; WOMAC = Western Ontario and McMaster Universities Osteoarthritis Index; SF = Short Form Health Survey; EQ-5D = EuroQol-5; KOOS = Knee Injury and Osteoarthritis Outcome Score

### Physical activity interventions that incorporate wearables

Three studies used a Fitbit Zip [34–36], one study used a Garmin Vivofit 2 [38], one study used Fitbit Flex or Fitbit One [37] and one study used a pedometer [39] for the intervention group. Five studies provided additional intervention components. These components included motivational interviewing (*n* = 1) [35], goal setting (*n* = 2) [34, 36], a tailored exercise program (*n* = 2) [34, 37], financial incentives [35] (*n* = 1), daily step goal sheet [38] (*n* = 1) and 12-week step goal sheet (*n* = 1) [39] (Table 1). Interventions were delivered in-person by a licensed physical therapist (*n* = 1) [34] or researcher (n = 1) [38], or remotely delivered by a health coach (*n* = 1) [35], or exercise physiologist (*n* = 1) [37]. Two studies [36, 39] used a hybrid format, which included 12 weekly action planning phone calls by researcher and 3 monthly in-person group support meetings in community [36] or 12 weekly phone calls and 3 in-person assessment visits [39]. Intervention duration ranged from 6 weeks to 6 months (Table 1). The wearable-based interventions were delivered immediately after surgery [38] or over an average of 14 days [34], 3 weeks [39], or 6-to 8-weeks [36] after orthopedic surgery. In-person sessions involved a single visit [38], 3 visits [39] or were dependent on the number of physical therapy visits [34]. For interventions delivered remotely, the number of phone calls ranged from 12 to 16 and were administered on weekly or biweekly basis [35–37, 39]. One intervention had 12 weekly phone calls as well as 3 monthly in-person group meetings [36]. Another intervention consisted of 14 calls, of which 4 calls were administered weekly and the remaining calls were administered on a biweekly basis [35]. No serious adverse events related to the use of wearables were reported by any of the studies that were included in this review.

### Comparison groups

Out of the six studies [34–39], one study provided the comparison group with wearable technology. However, participants did not receive feedback on their steps, steps progression, or counseling on physical activity goals [38]. Participants received in-person rehabilitation by licensed physical therapists (*n* = 1) [34], 3 face-to-face assessment visits under supervision of nurse (*n* = 1) [39], general information on recovery and rehabilitation via phone calls by research staff (*n* = 1) [35], weekly phone calls to assess health status by researcher (*n* = 1) [36], or 16-weeks of a tailored home-based exercise program based on American College of Sports Medicine guidelines (*n* = 1) [37].

### Outcome measurement

Four (67%) studies assessed physical activity as an outcome in both intervention and control groups, while one study (17%) assessed physical activity only in the intervention group. These four (67%) studies quantified physical activity as steps per day using an accelerometer such as the Actigraph GT3X [34, 36], Fitbit [35], or Garmin Vivofit 2 [38]. Steps per day were assessed at baseline (ranged from 14 days to 8 weeks after surgery), 6 months after surgery, and 6 and 12 months after discharge from physical therapy. Two studies quantified physical activity as time spent in MVPA using an Actigraph GT3X [34, 35]. Time in MVPA was assessed at baseline (on average 14 days after surgery), 6 months after surgery, and 6 and 12 months after discharge from physical therapy. One study quantified physical activity as steps per day, walking distance, and walking time using a pedometer at 1-, 2-, and 3-months after surgery in participants who received the physical activity intervention.

Performance-based measures such as 6-MWT, 4-meter walk test, and TUG were conducted by two (40%) [36, 37], one (20%) [36], and one (20%) [36] studies, respectively, to objectively quantify physical function. One (20%) study used the KOOS [38] and one (20%) study used the WOMAC [37] to assess patient-reported physical function. Physical function assessment using performance-based or patient-reported measures were conducted between 6 to 8 weeks and 4 to 6 months after surgery, respectively.

Pain was assessed with the EQ-5D and KOOS at the preoperative visit and 6 months after surgery in one study (17%) [38] and using the McGill Pain Questionnaire (*n* = 1) at the preoperative visit and 3-weeks, 1-, 2-, and 3-months after surgery in one study (17%) [39]. Psychological distress (i.e., anxiety/depression) was assessed at the preoperative visit and 6 months after surgery via one item from the EQ-5D in one study (17%) [38]. At preoperative visit and 3-weeks, 1-, 2-, and 3-months after surgery, disability was assessed using Oswestry Disability Index in one study (17%) [39]. General health was assessed through the SF-36 in two studies [37, 39] and EQ-5D [38] at a preoperative visit, or 6 to 8 weeks, and 4 to 6 months after surgery.

### Risk of bias

The PEDro scores of the studies included-in the review ranged from 2 to 6 (Table 2). Three studies [34, 35, 38] were moderate quality (PEDro score of 5 to 6) and the others [36–39] were poor quality (PEDro score < 5). All trials used random allocation, reported between group comparisons, and had similar group characteristics at baseline. Concealed allocation was performed in one study [34]. Owing to the nature of the interventions, participant and/or therapist blinding was not possible. Assessor blinding was noted in one study [34]. In addition, one study included an intention-to-treat analysis [35] and four studies reported points and estimates of variability [34–36, 39].

**Table 2:** Risk of bias of included studies (PEDro).

Study name	Eligibility criteria specified	Random allocation	Concealed allocation	Groups similar at baseline	Participant blinding	Therapist blinding	Assessor blinding	Adequate follow-up	Intent to treat analysis	Between group comparisons	Point estimates and variability	Total (0–10)
Aldemir et al, 2021 [39]	1	1	0	1	0	0	0	0	0	1	1	4
Christiansen et al, 2020 [34]	1	1	1	1	0	0	1	0	0	1	1	6
Losina et al, 2018 [35]	1	1	0	1	0	0	0	0	1	1	1	5
Paxton et al, 2018 [36]	0	1	0	1	0	0	0	0	0	0	1	3
Smith et al, 2019 [37]	0	1	0	1	0	0	0	0	0	0	0	2
Van der Walt et al, 2018 [38]	1	1	0	1	0	0	0	1	0	1	1	5

Note: Scoring of eligibility criteria specified does not contribute to total score

### Summary of findings: Effects on physical activity

Five (83%) studies [34–36, 38, 39], three moderate quality and two poor quality, reported within-group improvement in steps per day following a physical activity intervention using wearables. One poor quality study [39] assessing patients undergoing lumbar disc herniation surgery failed to assess physical activity in the control group, so between-group improvements could not be assessed; however, this study reported within-group improvement in walking time and distance within the intervention group. The three moderate quality studies [34, 35, 38] showed significantly greater between-group improvement in steps per day compared to the control group in adults after total knee or hip arthroplasty. Specifically, Christiansen et al. [34] showed participants, who underwent total knee arthroplasty and received an in-person physical activity intervention that used wearables and was delivered by a licensed physical therapist, walked 1,798 (95% confidence interval = 240 to 3,355) and 1,945 (95% confidence interval = 466 to 3,422) more steps per day at 6 and 12 months post discharge from physical therapy, respectively, compared to those who received usual care [34]. In another study, participants who underwent total knee arthroplasty and received feedback from wearable technology in addition to remote counseling by health coach and financial incentives walked on average 1,128 (95% confidence interval = 14 to 2,241) more steps per day compared to a usual care group at 6 months after surgery [35]. Van der Walt et al. [38] compared steps per day between an intervention group who received feedback from wearables and goals vs. control group (without feedback and step goals) and found that participants who underwent total knee or hip arthroplasty and received the intervention walked, on average, 656 (p = 0.005) and 570 (p = 0.030) more steps per day at 6 weeks and 6 months after surgery, respectively. This effect was further quantified using Cohen’s d, which ranged from 0.4 to 0.5.

Two (40%) of the moderate quality studies [34, 35] reported within-group improvements in time spent in MVPA [34, 35] after physical activity interventions that used wearables in adults after total knee arthroplasty (Table 3). However, the between-group intervention effects on MVPA were conflicting. At 6 months after surgery or discharge from physical therapy, time spent in MVPA was not statistically different compared to a usual care [34] or control group [35]. However, Christiansen et al. [34] found that participants who received a physical activity intervention spent 76 minutes (95% confidence interval = 10, 141) more per week in MVPA compared to usual care group (p < 0.05) at 12 months post-discharge from physical therapy [34]. Participants in the intervention group also on average spent the time in MVPA as recommended by Physical Activity Guidelines [10] at 6 months after discharge from physical therapy.

**Table 3:** Effects of physical activity interventions that use wearables on outcomes related to physical activity in adults after orthopaedic surgery.

Outcomes	Study name	Mean ± SD (95%CI)		Mean (95%CI) or p-values
		Intervention group	Control group	Between Group differences
**Steps per day (crude values)**				
Preoperative visit	Van der Walt et al, 2018 [38]	6953	7655	0.146
Baseline (average 14 days after surgery)	Christiansen et al 2020 [34]	2494±1391 (1803, 3186)	2214±1407 (1573, 2855)	280 (–631, 1191)
Baseline (after surgery)^	Losina et al 2018 [35]	5229 (4355, 6103)	6158 (5320, 6995)	NR
Baseline (6 to 8 weeks after surgery)	Paxton et al 2018 [36]	5754±2714 (4620, 6888)	5011±2038 (4129, 5893)	743 (-696, 2182)
1 month after surgery	Aldemir et al, 2021 [39]	3204.24±1131.48	NR	NR
6 weeks after surgery	Van der Walt et al, 2018 [38]	7162	6506	0.005
2 month after surgery	Aldemir et al, 2021 [39]	4959.98±1755.71	NR	NR
3 month after surgery	Aldemir et al, 2021 [39]	5254.02±1912.42	NR	NR
4.5 to 5 months after surgery	Paxton et al, 2018 [36]	6917±3445 (5783, 8052)	5291±2298 (4183, 6399)	1626 (-127, 3379),
6 months after surgery	Van der Walt et al, 2018 [38]	9526	8956	0.030
6 months post discharge from physical therapy	Christiansen et al, 2020 [34]	5739±2,665 (4369, 7109)	3941±1910 (3021, 4863)	1798 (240, 3,355)
6 months after surgery	Losina et al, 2018 [35]	7054 (5967, 8142)	6712 (5670, 7755)	1128 (14, 2241)^¥^
12 months post discharge from physical therapy	Christiansen et al, 2020 [34]	6114±1989 (4966, 7262)	4169±1890 (3123, 5217)	1945 (466, 3422)
**MVPA, minutes per week (crude values)**				
Baseline (average 14 days after surgery)	Christiansen et al, 2020 [34]	35.6±37.9 (16.7, 54.5)	19.4 ± 20.8 (9.9, 28.9)	16.2 (–3.7, 35.7)
Baseline (after surgery)^	Losina et al, 2018 [35]	10 (-1, 20)	22 (12, 33)	NR
6 months after surgery	Losina et al, 2018 [35]	49 (26, 72)	35 (13, 57)	25 (-4, 54)^¥^
6 months post discharge from physical therapy	Christiansen et al, 2020 [34]	150.6±161.2 (67.7, 233.5)	77.2±91.3 (33.3, 121.2)	73.4 (–14.1, 160.9)
12 months post discharge from physical therapy	Christiansen et al, 2020 [34]	133.8±98.1 (77.1, 190.4)	57.7±72.7 (17.5, 98.0)	76.1 (10.5, 141.5)

NR = not reported

^information on time after surgery for baseline assessment not available

^¥^change over 6 months adjusting for baseline scores

MVPA = moderate-to-vigorous physical activity

Van der Walt [38] provided the steps per day values at 6 weeks and 6 months after surgery as percent of preoperative steps per day. Therefore, percentage values were converted to crude average steps per day values by multiplying the percent by average preoperative steps per day.

### Summary of findings: Effects on physical function, pain, psychological distress and general health

Among participants undergoing total knee or hip arthroplasty, one poor quality study [36] reported minimal within-group improvements on 4-meter walk (walked 0.05 m/seconds faster), 6-MWT (walked 50 meters more) and TUG (took 0.94 seconds lesser) from 6–8 weeks to 4.5–5 months after surgery (Table 4). Two (40%) poor quality studies [36, 37] reported no significant between-group difference for 6-MWT and one (20%) poor quality [36] reported no significant between-group difference for 4-meter walk test and TUG at 4.5 to 5 months after surgery.

**Table 4:** Effects of physical activity interventions that use wearables on outcomes related to pain, physical function, general health and psychological distress in adults after orthopaedic surgery.

Outcomes	Study name	Mean±SD (95%CI)	
		Intervention group	Control group
**Pain**			
**Verbal Pain Severity (McGill Pain Questionnaire)** (score range from 0 to 5)			
Preoperative visit	Aldemir et al, 2021 [39]	4.24±0.75	4.18±0.93
3 weeks after surgery	Aldemir et al, 2021 [39]	0.79±0.69	1.06±0.85
1 month after surgery	Aldemir et al, 2021 [39]	0.61±0.60	0.88±0.88
2 month after surgery	Aldemir et al, 2021 [39]	0.55±0.66	1.06±0.88
3 month after surgery	Aldemir et al, 2021 [39]	0.55±0.71	0.79±1.00
**KOOS Pain** (score range from 0 to 100)			
Preoperative visit	Van der Walt et al, 2018 [38]	47.0±16.0	45.0±18.0
6 months after surgery	Van der Walt et al, 2018 [38]	86.0±13.8	85.4±15.3
**EQ-5D Pain** (score range from 0 to 5)			
Preoperative visit	Van der Walt et al, 2018 [38]	3.2±0.9	3.3±0.6
6 months after surgery	Van der Walt et al, 2018 [38]	1.7±0.8	1.8±0.7
**ODI: Disability** (score range from 0 to 100)			
Preoperative visit	Aldemir et al, 2021 [39]	37.09±6.41	36.91±8.36
3 weeks after surgery	Aldemir et al, 2021 [39]	11.15±5.30	13.65±7.36
1 month after surgery	Aldemir et al, 2021 [39]	7.76±4.55	9.62±7.63
2 month after surgery	Aldemir et al, 2021 [39]	5.09±5.35	9.06±8.439
3 month after surgery	Aldemir et al, 2021 [39]	3.45±5.04	6.65±6.26
**Physical function**			
**6 minute walk test, meters**			
Baseline (6 to 8 weeks after surgery)	Paxton et al, 2018 [36]	455±115 (408, 512)	466±98 (428, 516)
4.5 to 5 months after surgery	Paxton et al, 2018 [36]	510±122 (458, 574)	521±96 (480, 574)
**TUG, seconds**			
Baseline (6 to 8 weeks after surgery)	Paxton et al, 2018 [36]	9.17±2.77 (7.94, 10.34)	8.76±1.94 (7.91, 9.61)
4.5 to 5 months after surgery	Paxton et al, 2018 [36]	8.23±4.42 (7.10, 9.37)	8.09±1.91 (7.19, 8.98)
**Gait speed, 4 meter walk test (meters/second)**			
Baseline (6 to 8 weeks after surgery)	Paxton et al, 2018 [36]	1.66±0.33 (1.33, 1.98)	1.52±0.35 (1.18, 1.86)
4.5 to 5 months after surgery	Paxton et al, 2018 [36]	1.71±0.37 (1.35, 2.08)	1.61±0.54 (1.08, 2.14)
**KOOS Function** (score range from 0 to 100)			
Preoperative visit	Van der Walt et al, 2018 [38]	50.0±18.0	51.0±21.0
6 months post surgery	Van der Walt et al, 2018 [38]	87.3±10.2	86.4±13.7
**KOOS Quality of Life** (score range from 0 to 100)			
Preoperative visit	Van der Walt et al, 2018 [38]	30.0±19.0	33.0±18.0
6 months post surgery	Van der Walt et al, 2018 [38]	75.5±17.2	75.5±17.2
**EQ-5D Anxiety/depression** (score range from 0 to 5)			
Preoperative visit	Van der Walt et al, 2018 [38]	1.6±0.9	1.6±0.8
6 months post surgery	Van der Walt et al, 2018 [38]	1.2±0.6	1.3±0.6

ODI = Oswestry Disability Index; TUG = Time UP and Go Test; EQ-5D = EuroQol-5, total score of each subscale is 5; KOOS = Knee Injury and Osteoarthritis Outcome Score and total score of each subscale is 10

Smith et al. [37] presented data as pooled estimates; therefore, it was not possible to examine within-group changes in patient-reported measures of physical function and general health for participants who only received a physical activity intervention. One (20%) moderate quality study [38] reported within-group improvement in patient-reported measures of pain, physical function and general health for participants receiving feedback from wearables and step goals but no between-group differences were noted for these measures and for psychological distress from preoperative visit to 6 months after total knee or hip arthroplasty.

Among participants undergoing surgery for lumbar disc herniation, one study [39] reported within-group improvements in pain, disability, and quality of life that were assessed using questionnaires from preoperative visit to 3-weeks, 1-, 2- and 3-months after surgery. However, between group differences over time were not assessed in this study.

This systematic review provides summary evidence on the effects of interventions that used wearable technology as a primary component, either stand-alone or in combination with education or rehabilitation, in adults undergoing orthopaedic surgical procedures. Most studies included in this review were conducted in a total knee arthroplasty population, and one of the studies also included patients undergoing total knee or hip arthroplasty. Only one study included patients undergoing lumbar disc herniation surgery. The effects of physical activity interventions using wearables, either delivered in-person or remotely, appear promising for increasing steps per day. However, the study conducted in participants undergoing spine surgery did not measure physical activity in the control group; thus, between-group effects could not be determined. The between-group effects on MVPA and performance-based and patient-reported measures of physical function, pain, psychological distress, and general health were no different compared to a control group. None of the studies reported any adverse events related to wearable technology. The findings of this review suggest wearables may serve as a tool for healthcare professionals to promote physical activity following total knee or hip arthroplasty.

The use of wearables within a physical activity intervention can promote improvement in steps per day in adults following total knee or hip arthroplasty. This is an important finding as low levels of physical activity are often observed in patients after these procedures [5–7]. The beneficial effects on steps per day reported in our systematic review are consistent with previous literature showing that pairing wearables with step goals increases physical activity in adults with musculoskeletal disorders who were managed non-operatively [20, 40, 41]. One plausible explanation for this finding is that wearables can be used for real-time, patient-centered goal setting through the use of self-monitoring and feedback of daily steps [16, 42]. Taking more steps per day is known to improve health outcomes and reduce the risk of all-cause mortality [11, 43], which suggest that wearables may play an integral role in enhancing postoperative recovery following total knee or hip arthroplasty [44]. Notably, we did not find studies in orthopaedic surgery populations other than total joint arthroplasty or lumbar disc herniation. However, published conference abstracts [45] have shown that a wearable-based intervention, which included calibrated pedometers, telephonic counseling from a research personnel, education on physical activity, and walking goals had an effect in improving patient-reported physical activity at 6 and 12 months after spine surgery compared to a control group [45]. Thus, though wearables are a potential tool for promoting physical activity [46, 47], more evidence is needed on the benefits following spine surgery because evidence suggest that this surgical population demonstrate low physical activity levels that do not improve to a similar degree as other outcomes such as physical function [4, 5, 48].

Findings suggest that the effects on steps per day do not depend on whether the studied intervention was delivered in-person by a licensed physical therapist or remotely by a health coach. Traditionally, physical therapists target range of motion, pain, strength, and function during home and/or in-person sessions after surgery [49, 50]. The findings of this review suggest that physical activity may be added as a component of postoperative rehabilitation. Technological advancements and an evolving healthcare environment have shifted the delivery focus to remote and telehealth interventions to improve access and minimize barriers related to transportation [29]. Pairing wearable technology with remote goal setting and/or health coaching may be a complementary approach for healthcare professionals as part of in-person or telehealth visits. However, future work is needed to test the cost-effectiveness of this approach given it has the potential to shift the practice paradigm for postoperative rehabilitation for patients with musculoskeletal disorders.

Physical activity interventions use wearables did not show additional benefits compared to controls on clinical outcomes of physical function, pain, psychological distress, and general health measured at 6 months after surgery. Investigating the effects on function and pain after orthopaedic surgery is important given one in three adults fail to achieve clinical improvement in these postoperative outcomes [51–54]. Further, the presence of postoperative pain and psychological distress, such as depression, influence the recovery trajectory following orthopaedic surgery [55–58]. Our findings of lack of benefit on these outcomes are consistent with a recent meta-analysis of wearable-based interventions in adults with rheumatic diseases such as osteoarthritis, and rheumatic inflammatory diseases [21]. A systematic review led by Mansi et al. [20] reported significant within-group improvements in pain and disability in adults with musculoskeletal disorders managed non-operatively, however, the effects were not significant when compared with the control group. Future high-quality trials are needed to determine whether wearable technology can optimize the recovery trajectory for these relevant clinical outcomes following orthopaedic surgery.

This systematic review was prospectively registered and conducted in accordance with established guidelines, in terms of search strategy, study selection, and quality appraisal. Multiple authors were involved in the conduct of this review to ensure data accuracy and confidence in the results. We included only randomized controlled trials in the search protocol. However, two studies [34, 36] included in this review were feasibility trials. The findings of this review should be viewed in light of the limitations. First, we could not assess the theoretical models used to design the wearable-based interventions due to the lack of information reported in the included studies, such as models of behavioral change. Future research should focus on theoretical models to promote behavioral change through wearable technology in this patient population. Second, meta-analysis and examination of publication bias were not performed on the 5 eligible studies. At least 10 studies are needed to investigate publication bias [59]. The number of study participants was low and there was variability in the intervention duration, precluding the ability to perform meta-analysis. Lastly, the findings of this review on outcomes after orthopaedic surgery should be viewed with caution since none of the studies were rated as high quality on the PEDro scale (i.e., score ≥7) and there was a variability in intervention duration and timing post-surgery. Future high-quality clinical trials, which include comprehensive outcome assessment, blinding of research personnel involved with outcome assessment, and adequate follow-up and intent-to-treat analysis, are needed to investigate the efficacy of wearable-based physical activity interventions on objective and patient-reported outcomes in patients undergoing orthopaedic surgical procedures.

## Conclusions

Physical activity interventions that use wearables may have a positive impact on steps per day in patients after total knee or hip arthroplasty. However, this finding should be viewed with caution since it is based on 3 moderate quality studies. There was no clear evidence to conclude the effects of such interventions on MVPA, physical function, pain, psychological distress, and general health. Further high-quality research is needed to determine the potential benefit of wearable technology for the improvement of objective and patient-reported outcomes in patients undergoing orthopaedic surgical procedures.

## Supporting information

S1 Table Search strategy for PubMed database. (DOCX)

S2 Table Search strategy for Cumulative Index to Nursing and Allied Health Literature (CINAHL) database. (DOCX)

S3 Table Search strategy for PsycINFO database. (DOCX)

S4 Table Search strategy for EMBASE database. (DOCX)
