# Supplementary material for: Effects of physical activity interventions using wearables to improve objectively-measured and patient-reported outcomes in adults following orthopaedic surgical procedures: A systematic review
Source: PLoS One. 2022 Feb 15;17(2):e0263562. doi: 10.1371/journal.pone.0263562 (PMC8846530; doi:10.1371/journal.pone.0263562)
Supplement: S1 Table — (DOCX) [file pone.0263562.s001.docx]

**Supplemental Table 1: Search Strategy for PubMed database**

| **Query** | **Results** |
| --- | --- |
| wearable | 18,483 |
| wearable electronic devices [MeSH] | 14,534 |
| pedometer | 2,867 |
| fitbit | 882 |
| fitness track | 1,915 |
| fitness trackers [MeSH] | 847 |
| step track | 5,485 |
| ((((((wearable) OR (wearable electronic devices [MeSH])) OR (pedometer)) OR (fitbit)) OR (fitness track)) OR (fitness trackers [MeSH])) OR (step track) | 38,218 |
| sedentary | 38,558 |
| sedentary behavior [MeSH] | 11,346 |
| health | 5,414,908 |
| health [MeSH] | 394,573 |
| walking | 113,257 |
| walking [MeSH] | 59,420 |
| function | 14,499,037 |
| steps | 222,009 |
| physical activity | 608,538 |
| recover | 250,838 |
| recovery of function [MeSH] | 56,412 |
| disability | 388,047 |
| pain | 902,606 |
| accelerometer | 16,012 |
| acceleromet - Spellcheck off | 1 |
| accelerometry [MeSH] | 10,437 |
| (((((((((((((((sedentary) OR (sedentary behavior [MeSH])) OR (health)) OR (health [MeSH])) OR (walking)) OR (walking [MeSH])) OR (function)) OR (steps)) OR (physical activity)) OR (recover)) OR (recovery of function [MeSH])) OR (disability)) OR (pain)) OR (accelerometer)) OR (acceleromet)) OR (accelerometry [MeSH]) | 18,976,249 |
| education | 1,940,456 |
| exercise | 503,217 |
| exercise [MeSH] | 215,912 |
| exercise therapy [MeSH] | 56,073 |
| physiotherapy | 204,115 |
| physical therapy | 351,520 |
| physical therapy modalities [MeSH] | 163,665 |
| rehabilitation | 690,590 |
| rehabilitation [MeSH] | 324,777 |
| usual care | 36,613 |
| standard care | 489,543 |
| ((((((((((education) OR (exercise)) OR (exercise [MeSH])) OR (exercise therapy [MeSH])) OR (physiotherapy)) OR (physical therapy)) OR (physical therapy modalities [MeSH])) OR (rehabilitation)) OR (rehabilitation [MeSH])) OR (usual care)) OR (standard care) | 3,325,989 |
| hand | 512,437 |
| wrist | 49,579 |
| elbow | 47,216 |
| foot | 155,019 |
| ankle | 82,054 |
| shoulder | 92,171 |
| knee | 185,881 |
| hip | 173,752 |
| back | 251,229 |
| neck | 381,470 |
| spinal | 422,182 |
| spine | 261,486 |
| lower extremity | 204,097 |
| upper extremity | 191,828 |
| musculoskeletal | 102,976 |
| back [MeSH] | 21,650 |
| neck [MeSH] | 31,569 |
| lumbar vertebrae [MeSH] | 55,834 |
| cervical vertebrae [MeSH] | 41,246 |
| spine [MeSH] | 152,807 |
| lower extremity [MeSH] | 173,705 |
| upper extremity [MeSH] | 173,682 |
| musculoskeletal diseases [MeSH] | 1,130,567 |
| ((((((((((((((((((((((hand) OR (wrist)) OR (elbow)) OR (foot)) OR (ankle)) OR (shoulder)) OR (knee)) OR (hip)) OR (back)) OR (neck)) OR (spinal)) OR (spine)) OR (lower extremity)) OR (upper extremity)) OR (musculoskeletal)) OR (back [MeSH])) OR (neck [MeSH])) OR (lumbar vertebrae [MeSH])) OR (cervical vertebrae [MeSH])) OR (spine [MeSH])) OR (lower extremity [MeSH])) OR (upper extremity [MeSH])) OR (musculoskeletal diseases [MeSH]) | 2,993,531 |
| replacement | 524,734 |
| arthroplasty | 103,908 |
| fusion | 316,841 |
| operative | 5,664,289 |
| surgical | 3,995,775 |
| surgery | 5,096,891 |
| orthopedic procedures [MeSH] | 328,250 |
| surgical procedures, operative [MeSH] | 3,304,052 |
| (((((((replacement) OR (arthroplasty)) OR (fusion)) OR (operative)) OR (surgical)) OR (surgery)) OR (orthopedic procedures [MeSH])) OR (surgical procedures, operative [MeSH]) | 6,484,673 |
| (((((((((((((((((((((((hand) OR (wrist)) OR (elbow)) OR (foot)) OR (ankle)) OR (shoulder)) OR (knee)) OR (hip)) OR (back)) OR (neck)) OR (spinal)) OR (spine)) OR (lower extremity)) OR (upper extremity)) OR (musculoskeletal)) OR (back [MeSH])) OR (neck [MeSH])) OR (lumbar vertebrae [MeSH])) OR (cervical vertebrae [MeSH])) OR (spine [MeSH])) OR (lower extremity [MeSH])) OR (upper extremity [MeSH])) OR (musculoskeletal diseases [MeSH]))  AND  ((((((((replacement) OR (arthroplasty)) OR (fusion)) OR (operative)) OR (surgical)) OR (surgery)) OR (orthopedic procedures [MeSH])) OR (surgical procedures, operative [MeSH])) | 1,183,894 |
| clinical trial [MeSH] | 362,328 |
| random allocation [MeSH] | 105,774 |
| method, single-blind [MeSH] | 30,758 |
| method, double-blind [MeSH] | 166,563 |
| randomized | 1,302,953 |
| controlled trial | 934,996 |
| controlled study | 983,216 |
| clinical trial | 1,265,061 |
| clinical study | 1,345,531 |
| feasibility | 368,992 |
| feasibility study [MeSH] | 75,046 |
| ((((((((((clinical trial [MeSH]) OR (random allocation [MeSH])) OR (method, single-blind [MeSH])) OR (method, double-blind [MeSH])) OR (randomized)) OR (controlled trial)) OR (controlled study)) OR (clinical trial)) OR (clinical study)) OR (feasibility)) OR (feasibility study [MeSH]) | 2,521,829 |
| ((((((((((wearable) OR (wearable electronic devices [MeSH])) OR (pedometer)) OR (fitbit)) OR (fitness track)) OR (fitness trackers [MeSH])) OR (step track))  AND  ((((((((((((((((sedentary) OR (sedentary behavior [MeSH])) OR (health)) OR (health [MeSH])) OR (walking)) OR (walking [MeSH])) OR (function)) OR (steps)) OR (physical activity)) OR (recover)) OR (recovery of function [MeSH])) OR (disability)) OR (pain)) OR (accelerometer)) OR (acceleromet)) OR (accelerometry [MeSH])))  AND  (((((((((((education) OR (exercise)) OR (exercise [MeSH])) OR (exercise therapy [MeSH])) OR (physiotherapy)) OR (physical therapy)) OR (physical therapy modalities [MeSH])) OR (rehabilitation)) OR (rehabilitation [MeSH])) OR (usual care)) OR (standard care)))  AND  ((((((((((((((((((((((((hand) OR (wrist)) OR (elbow)) OR (foot)) OR (ankle)) OR (shoulder)) OR (knee)) OR (hip)) OR (back)) OR (neck)) OR (spinal)) OR (spine)) OR (lower extremity)) OR (upper extremity)) OR (musculoskeletal)) OR (back [MeSH])) OR (neck [MeSH])) OR (lumbar vertebrae [MeSH])) OR (cervical vertebrae [MeSH])) OR (spine [MeSH])) OR (lower extremity [MeSH])) OR (upper extremity [MeSH])) OR (musculoskeletal diseases [MeSH])) AND ((((((((replacement) OR (arthroplasty)) OR (fusion)) OR (operative)) OR (surgical)) OR (surgery)) OR (orthopedic procedures [MeSH])) OR (surgical procedures, operative [MeSH]))))  AND  (((((((((((clinical trial [MeSH]) OR (random allocation [MeSH])) OR (method, single-blind [MeSH])) OR (method, double-blind [MeSH])) OR (randomized)) OR (controlled trial)) OR (controlled study)) OR (clinical trial)) OR (clinical study)) OR (feasibility)) OR (feasibility study [MeSH])) | 268 |
