# Supplementary material for: Effects of physical activity interventions using wearables to improve objectively-measured and patient-reported outcomes in adults following orthopaedic surgical procedures: A systematic review
Source: PLoS One. 2022 Feb 15;17(2):e0263562. doi: 10.1371/journal.pone.0263562 (PMC8846530; doi:10.1371/journal.pone.0263562)
Supplement: S2 Table — (DOCX) [file pone.0263562.s002.docx]

**Supplemental Table 2: Search Strategy for Cumulative Index to Nursing and Allied Health Literature (CINAHL) database**

| **#** | **Query** | **Results** |
| --- | --- | --- |
| S91 | S8 AND S28 AND S42 AND S78 AND S90 | 20 |
| S90 | S79 OR S80 OR S81 OR S82 OR S83 | 478892 |
| S89 | MW clinical trials | 179819 |
| S88 | MW random assignment | 69457 |
| S87 | MW single-blind studies | 15058 |
| S86 | MW double-blind studies | 51328 |
| S85 | MW pilot studies | 84730 |
| S84 | randomized | 268636 |
| S83 | clinical trial | 267179 |
| S82 | controlled trial | 203819 |
| S81 | controlled study | 19965 |
| S80 | clinical study | 46155 |
| S79 | feasibility | 52014 |
| S78 | S66 AND S77 | 185486 |
| S77 | S67 OR S68 OR S69 OR S70 OR S71 OR S72 OR S73 OR S74 OR S75 OR S76 | 761183 |
| S76 | MW arthroplasty | 42222 |
| S75 | MW spinal fusion | 10606 |
| S74 | MW orthopedic surgery | 18413 |
| S73 | MW surgery, operative | 27041 |
| S72 | surgery | 591053 |
| S71 | surgical | 258485 |
| S70 | operative | 79511 |
| S69 | fusion | 25049 |
| S68 | arthroplasty | 48749 |
| S67 | replacement | 88707 |
| S66 | S43 OR S44 OR S45 OR S46 OR S47 OR S48 OR S49 OR S50 OR S51 OR S52 OR S53 OR S54 OR S55 OR S56 OR S57 OR S58 OR S59 OR S60 OR S61 OR S62 OR S63 OR S64 OR S65 | 601905 |
| S65 | MW musculoskeletal diseases | 10341 |
| S64 | MW upper extremity | 8459 |
| S63 | MW lower extremity | 13987 |
| S62 | MW spine | 11204 |
| S61 | MW cervical vertebrae | 12861 |
| S60 | MW lumbar vertebrae | 18680 |
| S59 | MW neck | 33889 |
| S58 | MW back | 37288 |
| S57 | musculoskeletal | 35364 |
| S56 | upper extremity | 17580 |
| S55 | lower extremity | 32342 |
| S54 | spine | 45262 |
| S53 | spinal | 93895 |
| S52 | neck | 66409 |
| S51 | back | 0 |
| S50 | hip | 73947 |
| S49 | knee | 81285 |
| S48 | shoulder | 34942 |
| S47 | ankle | 33705 |
| S46 | foot | 54598 |
| S45 | elbow | 13362 |
| S44 | wrist | 13670 |
| S43 | hand | 93043 |
| S42 | S30 OR S31 OR S32 OR S33 OR S34 OR S35 OR S36 OR S37 OR S38 OR S39 OR S40 OR S41 | 1085689 |
| S41 | MW education | 563297 |
| S40 | MW exercise | 133784 |
| S39 | MW therapeutic exercise | 24932 |
| S38 | MW physical therapy | 52359 |
| S37 | MW rehabilitation | 148333 |
| S36 | standard care | 5332 |
| S35 | usual care | 10833 |
| S34 | rehabilitation | 189583 |
| S33 | physical therapy | 64240 |
| S32 | physiotherapy | 43888 |
| S31 | exercise | 195676 |
| S30 | education | 718897 |
| S29 | S9 OR S10 OR S11 OR S12 OR S13 OR S14 OR S15 OR S16 OR S17 OR S18 OR S19 OR S20 OR S21 OR S22 OR S23 OR S24 OR S25 OR S26 OR S27 | 2617869 |
| S28 | S9 OR S10 OR S11 OR S12 OR S13 OR S14 OR S15 OR S16 OR S17 OR S18 OR S19 OR S20 OR S21 OR S22 OR S23 OR S24 OR S25 OR S26 OR S27 | 2617869 |
| S27 | MW life style, sedentary | 8687 |
| S26 | MW health | 1414719 |
| S25 | MW walking | 24677 |
| S24 | MW step | 9142 |
| S23 | MW physical activity | 44592 |
| S22 | MW pain | 212004 |
| S21 | MW accelerometer | 0 |
| S20 | accelerometry | 5651 |
| S19 | accelerometer | 7040 |
| S18 | acceleromet | 1 |
| S17 | pain | 332390 |
| S16 | disability | 149636 |
| S15 | recover | 6720 |
| S14 | physical activity | 105391 |
| S13 | steps | 96490 |
| S12 | function | 318948 |
| S11 | walking | 42791 |
| S10 | health | 1918768 |
| S9 | sedentary | 16982 |
| S8 | (S1 OR S2 OR S3 OR S4 OR S5 OR S6 OR S7) | 7479 |
| S7 | MW wearable sensors | 2210 |
| S6 | MW pedometers | 1438 |
| S5 | MW fitness trackers | 244 |
| S4 | fitness track | 3 |
| S3 | fitbit | 420 |
| S2 | pedometer | 2342 |
| S1 | wearable | 4850 |
