# Supplementary material for: Effects of physical activity interventions using wearables to improve objectively-measured and patient-reported outcomes in adults following orthopaedic surgical procedures: A systematic review
Source: PLoS One. 2022 Feb 15;17(2):e0263562. doi: 10.1371/journal.pone.0263562 (PMC8846530; doi:10.1371/journal.pone.0263562)
Supplement: S3 Table — (DOCX) [file pone.0263562.s003.docx]

**Supplemental Table 3: Search Strategy for PsycINFO database**

| **Set #** | **Search** | **Results** |  |
| --- | --- | --- | --- |
| S1 | su(wearable devices) | 524 |  |
| S2 | wearable | 1,716 |  |
| S3 | pedometer | 901 |  |
| S4 | fitbit | 207 |  |
| S5 | fitness track | 164 |  |
| S6 | step track | 735 |  |
| S7 | su(wearable devices) OR wearable OR pedometer OR fitbit OR (fitness track) OR (step track) | 3,564 |  |
|  |  |  |  |
| S8 | sedentary | 8,251 |  |
| S9 | health | 1,388,790 |  |
| S10 | walking | 19,508 |  |
| S11 | function | 434,512 |  |
| S12 | steps | 94,152 |  |
| S13 | physical activity | 109,207 |  |
| S14 | recover | 8,269 |  |
| S15 | disability | 195,387 |  |
| S16 | pain | 117,745 |  |
| S17 | accelerometer | 3,153 |  |
| S18 | accelerometry | 1,626 |  |
| S19 | acceleromet | 1 |  |
| S22 | su(pain) | 72,943 |  |
| S23 | su(physical activity) | 40,198 |  |
| S24 | su(walking) | 9,274 |  |
| S25 | sedentary behavior | 5,441 |  |
| S26 | sedentary OR health OR walking OR function OR steps OR (physical activity) OR recover OR disability OR pain OR accelerometer OR accelerometry OR acceleromet OR su(pain) OR su(physical activity) OR su(walking) OR (sedentary behavior) | 1,983,410 |  |
|  |  |  |  |
| S27 | standard care | 34,349 |  |
| S28 | standard care | 17,423,636 |  |
| S29 | standard care | 34,349 |  |
| S31 | usual care | 10,386 |  |
| S32 | rehabilitation | 188,506 |  |
| S33 | physical therapy | 112,333 |  |
| S34 | physiotherapy | 7,257 |  |
| S35 | exercise | 93,897 |  |
| S36 | education | 771,905 |  |
| S37 | su(physical therapy) | 64,197 |  |
| S38 | su(physiotherapy) | 812 |  |
| S39 | su(exercise) | 40,293 |  |
| S40 | hand | 101,431 |  |
| S41 | (standard care) OR (standard care) OR (standard care) OR (usual care) OR rehabilitation OR (physical therapy) OR physiotherapy OR exercise OR education OR su(physical therapy) OR su(physiotherapy) OR su(exercise) | 18,281,921 |  |
|  |  |  |  |
| S42 | hand | 101,431 |  |
| S43 | wrist | 4,286 |  |
| S44 | elbow | 2,207 |  |
| S45 | foot | 11,827 |  |
| S46 | ankle | 2,783 |  |
| S47 | shoulder | 386,436 |  |
| S48 | knee | 5,238 |  |
| S49 | hip | 7,007 |  |
| S50 | back | 57,057 |  |
| S51 | neck | 11,249 |  |
| S52 | spine | 8,385 |  |
| S53 | spinal | 36,758 |  |
| S54 | lower extremity | 3,870 |  |
| S55 | upper extremity | 3,792 |  |
| S56 | musculoskeletal | 9,365 |  |
| S57 | su(musculoskeletal system) | 1,290 |  |
| S58 | hand OR wrist OR elbow OR foot OR ankle OR shoulder OR knee OR hip OR back OR neck OR spine OR spinal OR (lower extremity) OR (upper extremity) OR musculoskeletal OR su(musculoskeletal system) | 598,018 |  |
|  |  |  |  |
| S59 | su(operation (surgery)) | 147 |  |
| S60 | surgery | 55,382 |  |
| S61 | surgical | 31,265 |  |
| S62 | operative | 8,995 |  |
| S63 | fusion | 10,840 |  |
| S64 | arthroplasty | 747 |  |
| S65 | replacement | 14,309 |  |
| S66 | su(operation (surgery)) OR surgery OR surgical OR operative OR fusion OR arthroplasty OR replacement | 102,900 |  |
|  |  |  |  |
| S67 | (hand OR wrist OR elbow OR foot OR ankle OR shoulder OR knee OR hip OR back OR neck OR spine OR spinal OR (lower extremity) OR (upper extremity) OR musculoskeletal OR su(musculoskeletal system)) AND (su(operation (surgery)) OR surgery OR surgical OR operative OR fusion OR arthroplasty OR replacement) | 24,305 |  |
|  |  |  |  |
| S68 | su(clinical trials) | 53,394 |  |
| S69 | randomized | 96,186 |  |
| S70 | controlled trial | 65,999 |  |
| S71 | controlled study | 122,315 |  |
| S73 | clinical study | 697,993 |  |
| S74 | clinical trial | 110,845 |  |
| S75 | feasibility | 26,081 |  |
| S76 | su(clinical trials) OR randomized OR (controlled trial) OR (controlled study) OR (clinical study) OR (clinical trial) OR feasibility | 812,549 |  |
|  |  |  |  |
| S77 | (su(wearable devices) OR wearable OR pedometer OR fitbit OR (fitness track) OR (step track)) AND (sedentary OR health OR walking OR function OR steps OR (physical activity) OR recover OR disability OR pain OR accelerometer OR accelerometry OR acceleromet OR su(pain) OR su(physical activity) OR su(walking) OR (sedentary behavior)) AND ((standard care) OR (standard care) OR (standard care) OR (usual care) OR rehabilitation OR (physical therapy) OR physiotherapy OR exercise OR education OR su(physical therapy) OR su(physiotherapy) OR su(exercise)) AND ((hand OR wrist OR elbow OR foot OR ankle OR shoulder OR knee OR hip OR back OR neck OR spine OR spinal OR (lower extremity) OR (upper extremity) OR musculoskeletal OR su(musculoskeletal system)) AND (su(operation (surgery)) OR surgery OR surgical OR operative OR fusion OR arthroplasty OR replacement)) AND (su(clinical trials) OR randomized OR (controlled trial) OR (controlled study) OR (clinical study) OR (clinical trial) OR feasibility) | 10 |  |
|  |  |  |  |
