# Supplementary material for: Effects of physical activity interventions using wearables to improve objectively-measured and patient-reported outcomes in adults following orthopaedic surgical procedures: A systematic review
Source: PLoS One. 2022 Feb 15;17(2):e0263562. doi: 10.1371/journal.pone.0263562 (PMC8846530; doi:10.1371/journal.pone.0263562)
Supplement: S4 Table — (DOCX) [file pone.0263562.s004.docx]

**Supplemental Table 4: Search Strategy for EMBASE database**

| **Embase <1974 to 2021 August 23>** | | **Results** |
| --- | --- | --- |
| 1 | wearable computer/ | 722 |
| 2 | wearable.ab,kw,ti. | 18413 |
| 3 | wearable electronic devices.ab,kw,ti. | 322 |
| 4 | step tracking.ab,kw,ti. | 154 |
| 5 | pedometer.ab,kw,ti. | 3211 |
| 6 | fitbit.ab,kw,ti. | 1222 |
| 7 | fitness tracker.ab,kw,ti. | 166 |
| 8 | fitness tracking.ab,kw,ti. | 91 |
| 9 | step tracker.ab,kw,ti. | 3 |
| 10 | activity tracker/ | 1345 |
| 11 | 1 or 2 or 3 or 4 or 5 or 6 or 7 or 8 or 9 or 10 | 23492 |
| 12 | sedentary lifestyle/ | 16224 |
| 13 | health/ | 218631 |
| 14 | walking/ | 73079 |
| 15 | step count/ | 1028 |
| 16 | disability/ | 114872 |
| 17 | pain/ | 326897 |
| 18 | accelerometer/ | 13969 |
| 19 | accelerometry/ | 8359 |
| 20 | sedentary.ab,kw,ti. | 45608 |
| 21 | health.ab,kw,ti. | 2711387 |
| 22 | walking.ab,kw,ti. | 109265 |
| 23 | steps per day.ab,kw,ti. | 1581 |
| 24 | steps/day.ab,kw,ti. | 1663 |
| 25 | physical activity.ab,kw,ti. | 169284 |
| 26 | recovery of function.ab,kw,ti. | 4744 |
| 27 | disability.ab,kw,ti. | 252143 |
| 28 | pain.ab,kw,ti. | 1011766 |
| 29 | accelerometer.ab,kw,ti. | 15747 |
| 30 | accelerometry.ab,kw,ti. | 6429 |
| 31 | functioning.ab,kw,ti. | 263242 |
| 32 | functioning.ab,kw,ti. | 263242 |
| 33 | 12 or 13 or 14 or 15 or 16 or 17 or 18 or 19 or 20 or 21 or 22 or 23 or 24 or 25 or 26 or 27 or 28 or 29 or 30 or 31 | 4268606 |
| 34 | education/ | 435012 |
| 35 | exercise/ | 297277 |
| 36 | physiotherapy/ | 90910 |
| 37 | rehabilitation/ | 86482 |
| 38 | usual care.ab,kw,ti. | 26904 |
| 39 | standard care.ab,kw,ti. | 16751 |
| 40 | rehabilitation.ab,kw,ti. | 257069 |
| 41 | physical therapy.ab,kw,ti. | 32611 |
| 42 | physiotherapy.ab,kw,ti. | 37704 |
| 43 | exercise.ab,kw,ti. | 385085 |
| 44 | exercise therapy.ab,kw,ti. | 6235 |
| 45 | education.ab,kw,ti. | 678999 |
| 46 | 34 or 35 or 36 or 37 or 38 or 39 or 40 or 41 or 42 or 43 or 44 or 45 | 1668237 |
| 47 | hand.ab,kw,ti. | 540747 |
| 48 | wrist.ab,kw,ti. | 50656 |
| 49 | elbow.ab,kw,ti. | 42379 |
| 50 | foot.ab,kw,ti. | 132333 |
| 51 | ankle.ab,kw,ti. | 84445 |
| 52 | shoulder.ab,kw,ti. | 89821 |
| 53 | knee.ab,kw,ti. | 199082 |
| 54 | hip.ab,kw,ti. | 195289 |
| 55 | back.ab,kw,ti. | 294927 |
| 56 | neck.ab,kw,ti. | 320625 |
| 57 | spinal.ab,kw,ti. | 364932 |
| 58 | spine.ab,kw,ti. | 183707 |
| 59 | lower extremity.ab,kw,ti. | 55122 |
| 60 | upper extremity.ab,kw,ti. | 30648 |
| 61 | musculoskeletal.ab,kw,ti. | 81766 |
| 62 | musculoskeletal injury/ | 4357 |
| 63 | 47 or 48 or 49 or 50 or 51 or 52 or 53 or 54 or 55 or 56 or 57 or 58 or 59 or 60 or 61 or 62 | 2175283 |
| 64 | hip replacement/ or replacement arthroplasty/ or total hip replacement/ or knee replacement/ | 15680 |
| 65 | surgery/ or orthopedic surgery/ | 645999 |
| 66 | replacement.ab,kw,ti. | 362805 |
| 67 | arthroplasty.ab,kw,ti. | 84972 |
| 68 | fusion.ab,kw,ti. | 261792 |
| 69 | operative.ab,kw,ti. | 438309 |
| 70 | surgical.ab,kw,ti. | 1395832 |
| 71 | surgery.ab,kw,ti. | 1765408 |
| 72 | 64 or 65 or 66 or 67 or 68 or 69 or 70 or 71 | 3391344 |
| 73 | 63 and 72 | 553723 |
| 74 | clinical study.ab,kw,ti. | 77046 |
| 75 | clinical trial.ab,kw,ti. | 248319 |
| 76 | controlled trial.ab,kw,ti. | 199953 |
| 77 | controlled study.ab,kw,ti. | 65626 |
| 78 | randomized.ab,kw,ti. | 833189 |
| 79 | feasibility.ab,kw,ti. | 285403 |
| 80 | feasibility study/ | 152023 |
| 81 | clinical trial/ | 1011721 |
| 82 | controlled study/ | 8326864 |
| 83 | 74 or 75 or 76 or 77 or 78 or 79 or 80 or 81 or 82 | 9465710 |
| 84 | 11 and 33 and 46 and 73 and 83 | 75 |
| 85 | limit 84 to (english and (article or article in press)) | 36 |
